# Synergistic Power of Piceatannol and/or Vitamin D in Bleomycin-Induced Pulmonary Fibrosis In Vivo: A Preliminary Study

**DOI:** 10.3390/biomedicines11102647

**Published:** 2023-09-27

**Authors:** Nehal Ezz Eldeen, Yasser M. Moustafa, Maha Abdullah Alwaili, Amani A. Alrehaili, Dina M. Khodeer

**Affiliations:** 1 Department of Pharmacology and Toxicology, Faculty of Pharmacy, Suez Canal University, Ismailia 41522, Egypt; 2 Pharmacology and Toxicology Department, Faculty of Pharmacy, Badr University in Cairo (BUC), Badr City, Cairo 11829, Egypt; 3 Department of Biology, College of Science, Princess Nourah bint Abdulrahman University, P.O. Box 84428, Riyadh 11671, Saudi Arabia; 4 Department of Clinical Laboratory Sciences, College of Applied Medical Sciences, Taif University, P.O. Box 11099, Taif 21944, Saudi Arabia

**Keywords:** piceatannol, vitamin D, HDAC2, HDAC4, PI3K/AKT, TGF-β, oxidative stress, bleomycin, pulmonary fibrosis

## Abstract

Oxidative stress and epigenetic alterations, including the overexpression of all class I and II histone deacetylases (HDACs), particularly HDAC2 and HDAC4, have been identified as key molecular mechanisms driving pulmonary fibrosis. Treatment with piceatannol (PIC) or vitamin D (Vit D) has previously exhibited mitigating impacts in pulmonary fibrosis models. The present study investigated the effects of PIC, Vit D, or a combination (PIC-Vit D) on the expression of HDAC2, HDAC4, and transforming growth factor-beta (TGF-β) in the lungs; the phosphatidylinositide-3-kinase (PI3K)/AKT signaling pathway; and the antioxidant status of the lungs. The objective was to determine if the treatments had protective mechanisms against pulmonary fibrosis caused by bleomycin (BLM) in rats. Adult male albino rats were given a single intratracheal dosage of BLM (10 mg/kg) to induce pulmonary fibrosis. PIC (15 mg/kg/day, oral (p.o.)), Vit D (0.5 μg/kg/day, intraperitoneal (i.p.)), or PIC-Vit D (15 mg/kg/day, p.o. plus 0.5 μg/kg/day, i.p.) were given the day following BLM instillation and maintained for 14 days. The results showed that PIC, Vit D, and PIC-Vit D significantly improved the histopathological sections; downregulated the expression of HDAC2, HDAC4, and TGF-β in the lungs; inhibited the PI3K/AKT signaling pathway; decreased extracellular matrix (ECM) deposition including collagen type I and alpha smooth muscle actin (α-SMA); and increased the antioxidant capacity of the lungs by increasing the levels of glutathione (GSH) that had been reduced and decreasing the levels of malondialdehyde (MDA) compared with the BLM group at a *p*-value less than 0.05. The concomitant administration of PIC and Vit D had a synergistic impact that was greater than the impact of monotherapy with either PIC or Vit D. PIC, Vit D, and PIC-Vit D exhibited a notable protective effect through their antioxidant effects, modulation of the expression of HDAC2, HDAC4, and TGF-β in the lungs, and suppression of the PI3K/AKT signaling pathway.

## 1. Introduction

Pulmonary fibrosis is a wound-healing reaction to lung damage. Chronic injury results in this abnormal wound-healing response, which leads to scarring. Eventually, scar tissue replaces the normal epithelial cells in the lungs, resulting in the formation of fibrotic tissue [1].

In 2019, a previously unknown coronavirus disease (COVID-19) spread globally, leading to tens of thousands of deaths and significant lung fibrosis in infected patients. Unfortunately, no definitive cure for the disease is currently available. Therefore, finding novel compounds to ameliorate or cure the associated pulmonary fibrosis would be valuable [2].

In response to injurious factors such as chronic infections, reactive oxygen species (ROS), some autoimmune disorders, chemical assaults, radiation, environmental toxins, and allergy reactions, alveolar epithelial cells release tissue factor (TF), which initiates the coagulation process [3,4]. The injured epithelial cells then release inflammatory mediators that induce the infiltration of inflammatory cells [5,6].These inflammatory cells release excessive cytokines and chemokines [6]. Myofibroblasts are contractile protein-expressing cells that may originate from fibroblast activation, migration, and differentiation that is triggered by cytokines and growth factors including mainly TGF-β, settled mesenchymal cells, bone marrow progenitors (also known as circulating fibrocytes), or resident epithelial cells undergoing epithelial-to-mesenchymal transitions (EMTs) and replacement with myofibroblasts in fibroblastic foci [6]. Myofibroblasts secrete extracellular matrix (ECM) proteins, including collagen type I and alpha smooth muscle actin (α-SMA) [7]. Excessive ECM protein synthesis and deposition without remodeling result in permanent damage to the lung architecture and compromised pulmonary function [7].

Furthermore, many studies have shown that there is a relationship between epigenetic alternations such as histone modification and the development of liver, kidney, and cardiac fibrosis. Histone acetylation is the most prevalent type of histone modification. It is controlled by two enzymes, histone acetyltransferases (HATs) and histone deacetylases (HDACs). HDACs deacetylate both histone and nonhistone proteins, enhance chromatin condensation, and suppress the gene expression that is responsible for apoptosis, cell cycle control, DNA repair, and metabolism [8]. Many studies have reported overexpression of all Class I and II HDACs, especially HDAC2 and HDAC4, in pulmonary fibrosis [8]. Furthermore, HDACs stimulate signal transducers and activators in the transcription 3 (STAT3) pathway, which increases the expression of ECM protein-coding genes such as collagen I and α-SMA [9,10]. Moreover, HDACs increase the proliferation of fibroblasts into myofibroblasts through the stimulation of the PI3K/AKT signaling pathway [9,10]. TGF-β is a crucial driver in the pathophysiology of fibrosis. It also regulates a number of physiological processes, such as cell proliferation, wound healing, immunity, and carcinogenesis [11].

Piceatannol (PIC) (3,5,3′,4′-trans-tetrahydroxystilbene) is a naturally hydroxylated analogue of resveratrol, which is found in grapes and red wine [11]. It has anti-inflammatory, anticancer, and antioxidant activities [12,13,14,15]. Several studies have shown that PIC has antifibrotic activity in renal and liver fibrosis through its downregulation of class I and II HDACs, and this resulted in decreased ECM protein production and inhibition of fibroblasts from becoming apoptosis-resistant myofibroblasts [7,11].

**Scheme 1:**
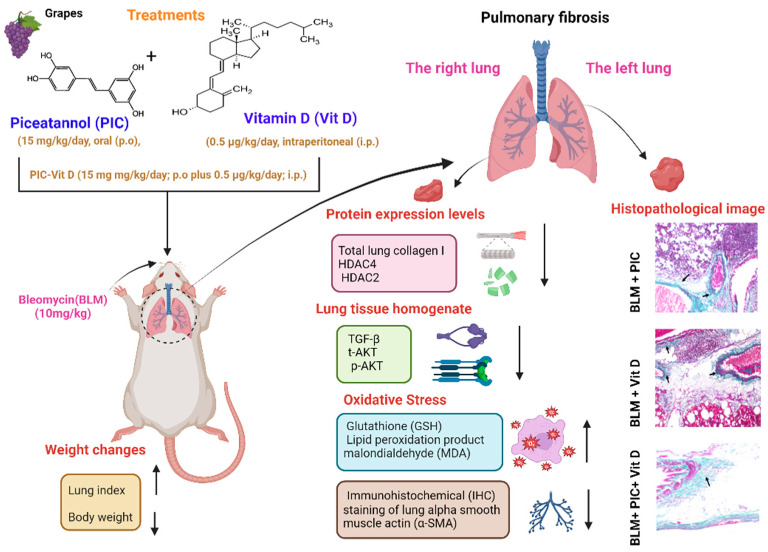
Illustration of the study’s experimental design, including the treatment regimen and parameters investigated.

Vitamin D (Vit D) is an important lipid-soluble vitamin that is associated with cell proliferation and differentiation, apoptosis, oxidative stress, matrix homeostasis, intercellular adhesion, and regulation of the inflammatory response [16]. Its deficiency is associated with numerous pulmonary diseases, including interstitial lung disease, pulmonary infections, chronic obstructive pulmonary disease, and cystic fibrosis [17]. There is evidence suggesting a correlation between Vit D deficiency, as well as deficiencies in other vitamins such as A, B, C, and E, and an increased susceptibility to pulmonary infections, including tuberculosis. Multiple studies have demonstrated that the administration of these vitamins resulted in a favorable impact on the prevention and management of tuberculosis [18,19]. Vit D exerts its antifibrotic activity by suppressing tissue factor (TF) expression, increasing the expression of the tissue factor pathway inhibitor (TFPI), decreasing the expression of inflammatory cytokines and growth factors, inhibiting the TGF-β-SMAD signaling pathway, and negatively regulating the renin-angiotensin system (RAS) [16]. The single effects of PIC or Vit D have been previously confirmed for their ameliorative effects on pulmonary infections. Our work will be the first to study their combined effect on the expression of histone deacetylases genes. The goals of the current study were to study the protective effects of PIC, Vit D, and a combination of the two (PIC-Vit D) in pulmonary fibrosis induced by bleomycin (BLM) in rats and the underling molecular pathways involved (Scheme 1).

## 2. Materials and Methods

### 2.1. Experimental Animals

The current study utilized 48 male albino Wistar rats aged 7 weeks and weighing between 150 and 220 g. The rats were acquired from a holding company for biological products and vaccines (Vacsera Co., Cairo, Egypt). The rats were housed in cages made of polyethylene, with each cage containing eight rats in conventional conditions (normal light/dark cycles, relative humidity of 55%, and temperature of 25 ± 3 °C) with free access to food and water. The rats were given a week to adapt to their new environment before the experiment was conducted. All the experimental procedures involving animals were sanctioned by the Animal Care and Use Committee at Suez Canal University’s Faculty of Pharmacy in Egypt (ethical number: 202011MA3).

### 2.2. Drugs and Chemicals

Bleomycin hydrochloride (Nippon Kayaku, Tokyo, Japan) was provided in vials containing 15 mg each and dissolved in phosphate buffered saline (PBS) at a pH of 7.4. Piceatannol powder (Career Henan Chemical Co., Zhengzhou City, Henan Province, China) was dissolved in dimethyl sulfoxide (DMSO) solvent. Vitamin D (Medical Union Pharmaceuticals, Ismailia, Egypt) was diluted with saline. The PBS and DMSO (Adwic Co., Cairo, Egypt) used in this study were of analytical quality.

### 2.3. Experimental Design

The rats were assigned into six groups at random. In group I, the rats were given a single intratracheal injection of PBS (BLM vehicle; 3.33 mL/kg) parallel to the BLM injection. In the second group, the rats were given a single intratracheal injection of BLM (10 mg/kg) to induce pulmonary fibrosis. The rats in group III were given a single intratracheal injection of BLM (10 mg/kg) followed by PIC (15 mg/kg/day/14 days, p.o.) [20,21] beginning from the next day of BLM injection in a volume of 100 mL/kg. The rats in group IV were given a single intratracheal injection of BLM (10 mg/kg) followed by Vit D (0.5 μg/kg/day/14 days, i.p.) [22] beginning from the next day of BLM injection. The rats in group V were given a single intratracheal injection of BLM (10 mg/kg) followed by PIC (15 mg/kg/day/14 days, p.o.) and Vit D (0.5 μg/kg/day/14 days, i.p.) beginning from the next day of BLM injection. The rats in group VI were given a single intratracheal dose of PBS (3 mL/kg) followed by PIC (15 mg/kg/day/14 days, p.o.) beginning from the next day of PBS injection. This group was made to investigate whether PIC had any toxic effects on lung tissues. The experiment lasted up to 14 days. The BLM was dissolved in PBS (3 mg/mL) and given as a single intratracheal dose, PIC powder was dissolved in DMSO (10 mg/mL) [23] and given orally by gastric gavage, and Vit D was diluted in saline (2800 IU/100 mL). The animals were weighed before, during, and after the experiment.

### 2.4. Tissue Sampling

The rats were sacrificed at the end of the experiment, and their lungs were then removed and washed in ice-cold PBS. Next, the lungs were allocated; the right lung was maintained at −80 °C for the measurement of total lung collagen I, HDAC4, HDAC2, TGF-β, t-AKT, p-AKT, reduced glutathione (GSH), and lipid peroxidation product malondialdehyde (MDA). The left lung was preserved via immersion in a 10% phosphate-buffered paraformaldehyde solution for 18 h at pH 7.4 before being embedded in paraffin. The tissues were then cut to a thickness of 4 µm and dried overnight at 37 °C. The samples were then deparaffinized, rehydrated, and processed for histopathological and α-SMA immunohistochemistry staining.

### 2.5. Tissue Homogenate Preparation

Tissues from the right lung were collected, weighed, and homogenized in PBS at 4 °C using a polytron homogenizer. Then, the homogenized tissue suspension was centrifuged to remove any cellular components, and the supernatant was used to measure lung TGF-β, t-AKT, p-AKT, GSH, and MDA protein levels, as well as Western blotting analysis of HDAC2, HDAC4, and collagen type I [17].

### 2.6. Measurements of Lung TGF-β, t-AKT, p-AKT, GSH, and MDA Levels Using ELISA Kits

Lung tissue homogenate was analyzed for TGF-β, t-AKT, p-AKT, GSH, and MDA protein levels using an enzyme-linked immunosorbent assay (ELISA) reader (Stat Fax 2200, Awareness Technologies, Palm City, FL, USA). Rat ELISA kits were used for the detection of TGF-β (Bio Vision incorporated, Cambridge, UK), t-AKT, p-AKT (Abcam, Cambridge, UK), GSH (Bio Vision incorporated, Cambridge, UK), and MDA (Bio Vision incorporated, Cambridge, UK).

### 2.7. Western Blotting Analysis of HDAC2, HDAC4 and Collagen I

To measure the protein expression levels for HDAC2, HDAC4, and collagen I, part of the prepared tissue homogenate suspension was centrifuged for 20 min at 14,000× *g* at 4 °C, and the supernatant of the protein mixture was measured using a Bradford protein assay kit (SK3041) from Bio Basics Inc. (Markham, ON L3R 8T4, Canada). Samples of equal concentrations of protein were separated using a sodium dodecyl sulphate polyacrylamide gel depending on their molecular weight and transferred to a polyvinylidene fluoride (PDVF) membrane that was then blocked via incubation at room temperature in tris-buffered saline with Tween 20 (TBST) buffer containing 3% bovine serum albumin (BSA) for 1 h to prevent non-specific binding. The blocked membrane was incubated with primary antibodies against blotted HDAC2, HDAC4, collagen I, and β-actin proteins overnight at 4 °C. After that, the membrane was incubated at room temperature with secondary antibodies for 1 h. Finally, the protein bands were visualized by applying a chemiluminescent substrate (Clarity ^TM^ Western ECL substrate, Bio-Rad cat#170-5060). A ChemiDoc MP imager was used to capture chemiluminescence signals and measure the intensity of the target protein bands compared to β-actin (a housekeeping protein) [24].

### 2.8. Immunohistochemical Staining and Image Analysis for α-SMA

For immunohistochemical (IHC) staining of lung α-SMA, 4 μm-thick sections were cut from selected paraffin blocks, followed by deparaffinization, rehydration, and antigen retrieval in a microwave oven for 20 min in citric acid buffer (pH 6.0). Then, the sections were incubated with α-SMA mouse monoclonal antibodies (Labvision^®^, Fremont, CA 94538, USA) overnight at 4 °C. Staining with DAB (a chromogen) and Mayer’s hematoxylin (a counterstain) was performed after conjugation with a streptavidin–biotin–peroxidase complex. An expert pathologist examined the slides in the dark using a light microscope (Olympus CX21, Tokyo, Japan) at ×40 magnification. The optical density of lung α-SMA was measured using the imageJ program, which was developed by the National Institute of Health in the United States [25,26].

### 2.9. Histopathological Examination of the Lung Tissues

The tissues from the left lung were fixed in 10% formalin buffer, and the paraffin sections (4 μm) were further produced for histological examination. Hematoxylin and eosin (H&E) and Masson’s trichrome stains were used to visualize the fibrotic lesions and assess the degree of fibrosis [25]. These sections were interpreted by an independent pathologist using a light microscope (Olympus^®^ CX21, Tokyo, Japan) at ×40 magnification for the H&E staining and at ×10 magnification for the Masson’s trichrome staining. The lung tissue was evaluated according to the presence of the following factors: inflammatory cell infiltrations in the perivascular, peribronchiolar, and alveolar walls, epithelial desquamation, parenchymal fibrosis, and emphysematous areas. Furthermore, the lung parenchyma was examined for a distorted appearance and impaired alveolar architecture [27].

### 2.10. Statistical Analysis

Statistical analysis of data was done using the statistical package for social sciences (SPSS) version 25 program. All the variables were described as mean ± standard error (SE). The specific variations between three or more group means were determined using a one-way analysis of variance (ANOVA) followed by Bonferroni’s post hoc test. Statistically significant variations were evaluated at *p* < 0.05.

## 3. Results

### 3.1. Effects of Piceatannol, Vitamin D, and a Combination of the Two on Lung TGF-β, t-AKT, and p-AKT Concentrations

**Figure 1:**
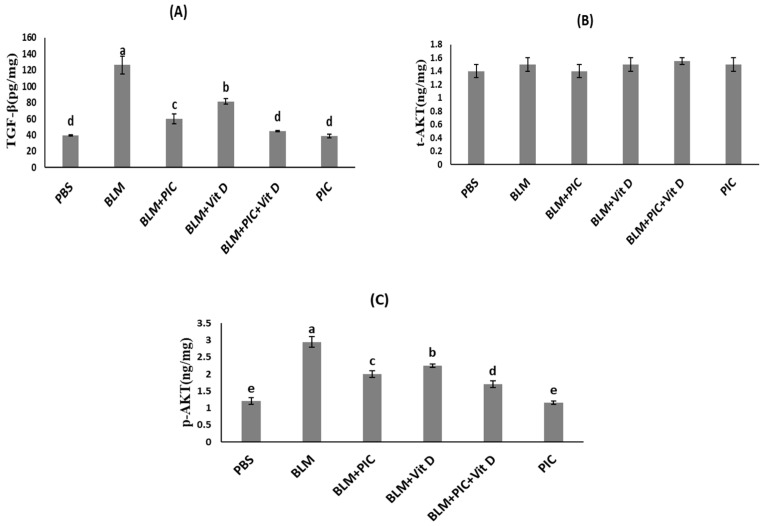
Effect of PIC, Vit D, or PIC-Vit D on lung tissue homogenate levels of (**A**) TGF-β, (**B**) t-AKT, and (**C**) p-AKT’ in BLM-induced pulmonary fibrosis in rats. Variables are described as mean ± SE and evaluated using a one-way ANOVA followed by Bonferroni’s post hoc test. PBS, phosphate buffered saline; BLM, Bleomycin; PIC, Piceatannol; Vit D, Vitamin D. The different letters indicate significant difference between different groups at *p* < 0.05, *n* = 8.

The concentration of t-AKT did not vary significantly between all the study groups (Figure 1B). Significant increases in the lung TGF-β and p-AKT concentrations were evaluated after BLM injection in comparison with the PBS group at *p* < 0.05 (Figure 1A,C). Treatment with PIC significantly decreased the concentration of TGF-β in comparison with the BLM group at *p* < 0.05 (Figure 1A,C). Treatment with Vit D significantly decreased the concentration of TGF-β in comparison with the group that received BLM alone (BLM group) and the BLM + PIC group. This treatment also significantly decreased the concentration of p-AKT in comparison with the BLM group but showed an insignificant change in the p-AKT concentration in comparison with the BLM + PIC group at *p* < 0.05 (Figure 1A,C). Coadministration of both PIC and Vit D was accompanied through a significant decrease in the TGF-β concentration in comparison with the BLM group and Vit D group, and through a significant reduction in the p-AKT concentration in comparison with the BLM, BLM + PIC and BLM + Vit D groups, showing an insignificant change in the TGF-β concentration in comparison with the BLM + PIC group at *p* < 0.05 (Figure 1A,C). The treatment of healthy rats with PIC led to an insignificant change in the TGF-β and p-AKT concentrations in comparison with the PBS group but a significant reduction in the TGF-β and p-AKT concentrations in comparison with the BLM group at *p* < 0.05 (Figure 1A,C). The combination group (BLM + PIC + Vit D) had the highest reductions in TGF-β and p-AKT concentrations of all the treated groups.

### 3.2. Effects of Piceatannol, Vitamin D, and a Combination of the Two on the Antioxidant Status of Lung Tissues

**Figure 2:**
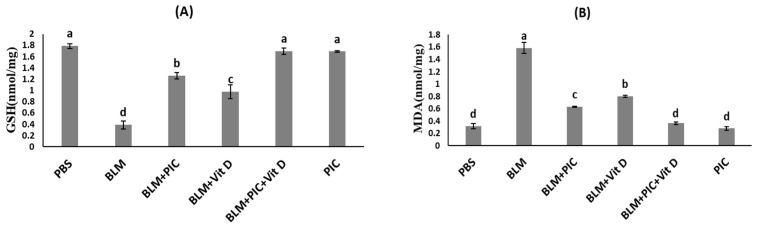
Effect of PIC, Vit D, or PIC-Vit D on the antioxidant status of lung tissue levels of (**A**) GSH, and (**B**) MDA in BLM-induced pulmonary fibrosis in rats. Variables are described as mean ± S.E. and evaluated using one-way ANOVA followed by Bonferroni’s post hoc test. PBS, phosphate buffered saline; BLM, Bleomycin; PIC, Piceatannol; Vit D, Vitamin D. The different letters indicate significant differences between different groups at *p* < 0.05, *n* = 8.

In this study, glutathione (GSH) and malondialdehyde (MDA) were also measured to assess the lung redox status. Statistically, a significant decrease in the GSH concentration and a significant increase in the MDA concentration were found after BLM injection in comparison with the PBS group at *p* < 0.05 (Figure 2A,B). Treatment with PIC significantly increased the GSH concentration but significantly decreased the MDA concentration in comparison with the BLM group at *p* < 0.05 (Figure 2A,B). Treatment with Vit D significantly increased the GSH concentration in comparison with the BLM group, showing a significantly lower concentration of GSH in comparison with the BLM + PIC group at *p* < 0.05 (Figure 2A,B). Treatment with Vit D significantly decreased the MDA concentration in comparison with the BLM group, showing a significantly higher concentration of MDA in comparison with the BLM + PIC group at *p* < 0.05 (Figure 2A,B). Coadministration of both PIC and Vit D was accompanied by a significant elevation in the GSH concentration in comparison with the BLM, BLM + PIC, and BLM + Vit D groups at *p* < 0.05 (Figure 2A,B). Treatment of healthy rats with PIC led to an insignificant change in the GSH and MDA concentrations in comparison with the PBS group but a significant elevation in the GSH concentration and a significant reduction in the MDA concentration in comparison with the BLM group at *p* < 0.05 (Figure 2A,B). The group treated with (BLM + PIC + Vit D) had the highest elevation in the GSH concentration and the highest reduction in the MDA concentration of all the treated groups (Figure 2A,B).

### 3.3. Effects of Piceatannol, Vitamin D, and a Combination of the Two on HDAC2, HDAC4, and Collagen I Concentrations in the Lungs

**Figure 3:**
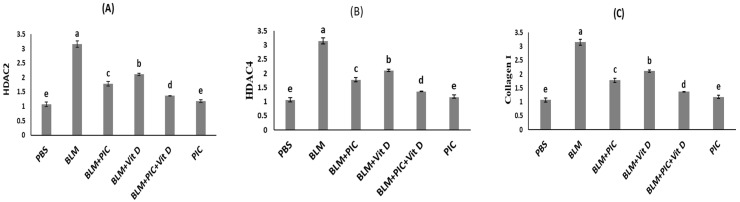

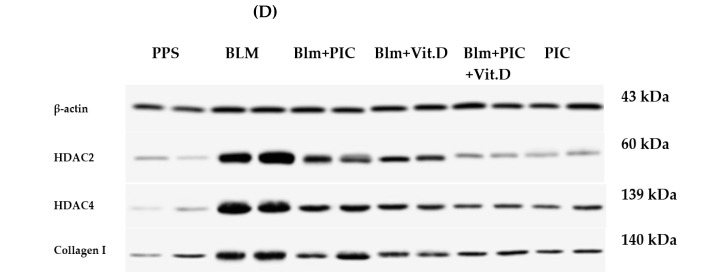
Effect of PIC, Vit D, or PIC-Vit D on lung tissue homogenate levels of (**A**) HDAC2, (**B**) HDAC4, (**C**) Collagen I, and (**D**) Western blotting analysis of BLM-induced pulmonary fibrosis in rats. Variables are described as mean ± SE and evaluated using a one-way ANOVA followed by Bonferroni’s post hoc test. PBS, phosphate buffered saline; BLM, Bleomycin; PIC, Piceatannol; Vit D, Vitamin D. The different letters indicate significant differences between different groups at *p* < 0.05, *n* = 8.

Significant elevations in the mean concentrations of HDAC2, HDAC4, and collagen I in the lungs were evaluated after BLM injection in comparison with the PBS group at *p* < 0.05 (Figure 3A–D). Treatment with PIC significantly decreased the concentrations of HDAC2, HDAC4, and collagen I in comparison with the BLM group at *p* < 0.05 (Figure 3A–D). Treatment with Vit D significantly decreased the concentrations of HDAC2, HDAC4, and collagen I in comparison with the BLM group and the BLM + PIC group at *p* < 0.05 (Figure 3A–D). Coadministration of both PIC and Vit D was accompanied by significant reductions in the HDAC2, HDAC4, and collagen I concentrations in comparison with the BLM, BLM + PIC, and BLM + PIC + Vit D groups at *p* < 0.05 (Figure 3A–D). Treatment of healthy rats with PIC led to an insignificant change in the HDAC2, HDAC4, and collagen I concentrations in comparison with the PBS group but significant reductions in the HDAC2, HDAC4, and collagen I concentrations in comparison with the BLM group at *p* < 0.05 (Figure 3A–D). The group treated with (BLM + PIC + Vit D) had the highest reductions in HDAC2, HDAC4, and collagen I concentrations of all the treated groups (Figure 3A–D).

### 3.4. Effects of Piceatannol, Vitamin D, and a Combination of the Two on Immunohistochemical Staining for α-SMA in Lung Tissue

**Figure 4:**
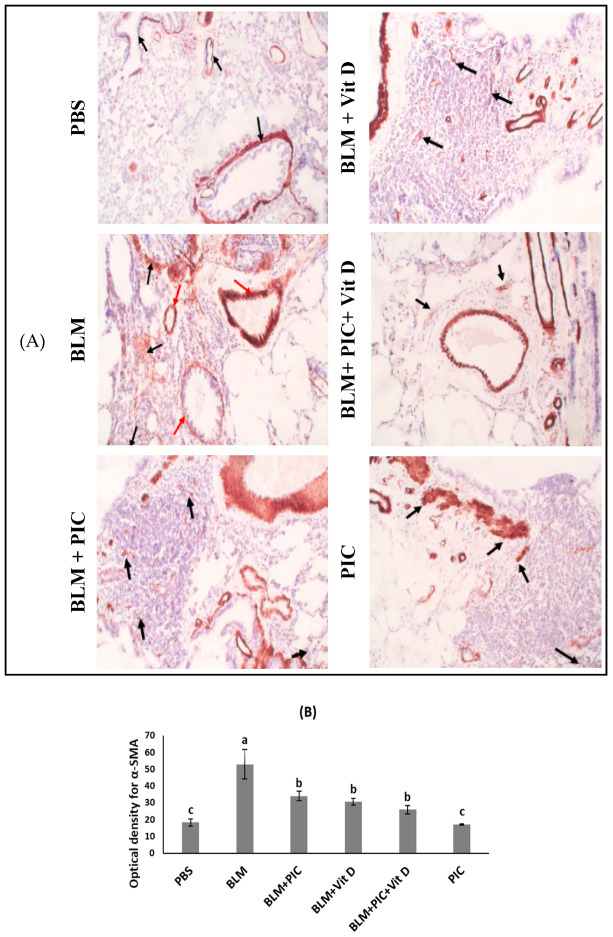
Effect of PIC, Vit D, or PIC-Vit D on (**A**) immunohistochemical staining of lung α-SMA (×20 magnification) and (**B**) optical density of α-SMA positive lung tissues in different study groups for BLM-induced pulmonary fibrosis in rats. Variables are described as mean ± SE and evaluated using a one-way ANOVA followed by Bonferroni’s post hoc test. PBS, phosphate buffered saline; BLM, Bleomycin; PIC, Piceatannol; Vit D, Vitamin D. The different letters indicate significant differences between different groups at *p* < 0.05, *n* = 8.

Immunohistochemical staining examinations showed a significant elevation in lung α-SMA immunostaining in the BLM group in comparison with the PBS group at *p* < 0.05 (Figure 4A,B). Treatment with PIC, Vit D, or PIC-Vit D significantly decreased the levels of α-SMA in the lungs in comparison with the BLM group at *p* < 0.05 (Figure 4A,B), showing insignificant changes between them. The treatment of healthy rats with PIC led to a significant reduction in α-SMA expressional levels in lung tissues in comparison with the BLM, BLM + PIC, and BLM + Vit D groups at *p* < 0.05 (Figure 4A,B), showing insignificant changes in the combination group (BLM + PIC + Vit D).

### 3.5. Effects of Piceatannol, Vitamin D, and a Combination of the Two on BLM-Induced Histopathological Changes

**Figure 5:**
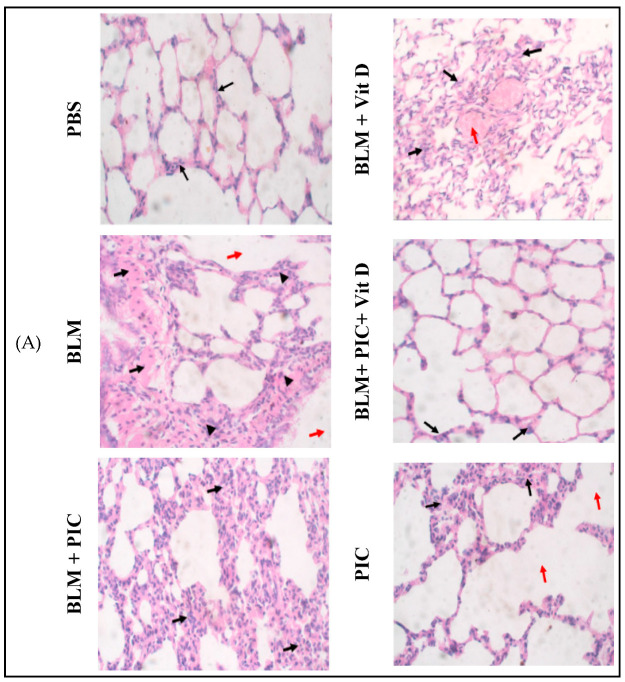

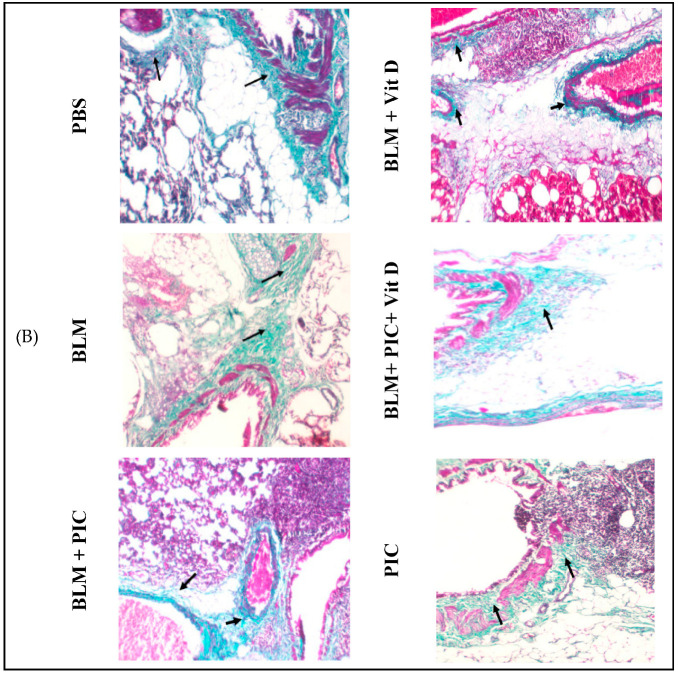
(**A**) Histopathological images of lung tissues of different study groups, H&E stain (×40). (**B**) Histopathological images of lung tissues of different study groups, Masson’s trichrome stain (×10). PBS; phosphate buffered saline, BLM; Bleomycin; PIC, Piceatannol; Vit D; Vitamin D.

Histological examination of the H&E sections of the lung tissues revealed that the PBS group showed normal alveolar and bronchial walls and uniform alveolar architecture (Figure 5A). Conversely, the BLM-treated group exhibited noticeable lung changes. Histological lung sections showed marked alveolar septa thickening, chronic pulmonary inflammation, leukocyte infiltration, and emphysematous areas with distortion of lung architecture (Figure 5A). The pulmonary damage induced by BLM was improved in all the treated groups. Treatment with PIC showed moderate thickening of the alveolar septa due to chronic inflammation and fibrosis (black arrows), and no emphysematous areas could be seen (Figure 5A). Treatment with Vit D showed that most of lung tissue showed uniform architecture (black arrows), while part of the lung tissue showed mild thickening of the alveolar septa dueto chronic inflammation and fibrosis (arrow heads) and congested blood vessels (red arrows). No emphysematous areas could be seen (Figure 5A). Co-administration of PIC and Vit D resulted in uniform alveolar architecture, thin alveolar walls, few chronic inflammatory cells, and congested blood vessels (black arrows) (Figure 5A). Treatment of healthy rats with PIC showed that most of the lung tissue showed uniform architecture (black arrows), while part of the lung tissue showed mild thickening of the alveolar septa due to chronic inflammation and fibrosis (arrow heads) and the presence of emphysematous areas (red arrows) (Figure 5A).

Masson trichome-stained sections of PBS showed normal alveolar walls and spaces without any fibrosis that could be detected (Figure 5B). Conversely, the BLM group showed marked expansion of the fibrous tissue (black arrows), especially in a peri-bronchiolar pattern (Figure 5B). The pulmonary fibrosis induced by BLM was improved in all the treated groups. Treatment with PIC resulted moderate peri-bronchiolar fibrous expansion (Figure 5B). Treatment with Vit D resulted in mild peri-bronchiolar fibrous expansion (Figure 5B). Co-administration of PIC and Vit D showed that most of the lung tissue showed uniform alveolar architecture and thin alveolar walls, while a small part of the lung tissue showed mild peri-bronchiolar fibrous expansion (Figure 5B). Treatment of healthy rats with PIC resulted in mild peri-bronchiolar fibrous expansion (Figure 5B).

## 4. Discussion

Pulmonary fibrosis is a lung interstitial disease with a 50% death rate within 3–5 years following diagnosis [28]. It has been identified as one of the most devastating complications of COVID-19; thus, the development of effective antifibrotic therapies with minimal adverse effects is urgently needed [29].

**Figure 6:**
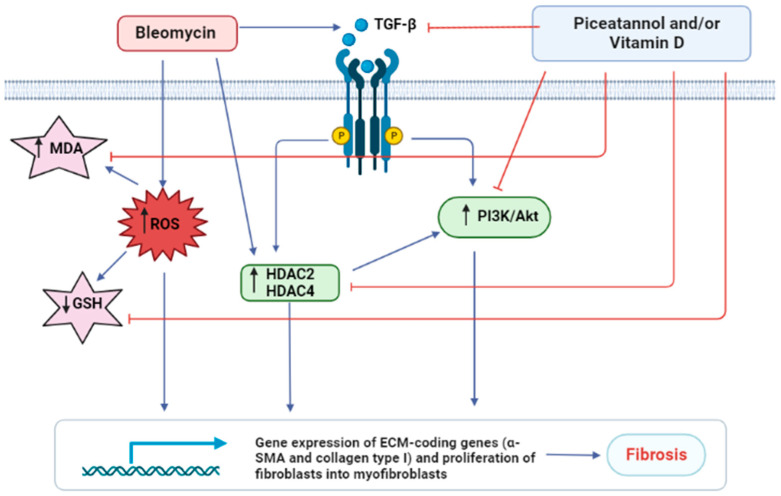
Possible mechanistic pathways of PIC, Vit D, or PIC-Vit D in protection against BLM-induced pulmonary fibrosis in rats. BLM increased the expression of TGF-β and MDA and decreased the expression of GSH. TGF-β increased the expressions of HDAC2 and HDAC4 and activated the PI3K/Akt signaling pathway, resulting in an increase in the expression of ECM proteins such as α-SMA and collagen type I, as well as the proliferation of fibroblasts into myofibroblasts. BLM may also increase HDAC2 and HDAC4 expression levels independently of TGF-β. HDAC2 and HDAC4 aided TGF-β in the activation of the PI3K-Akt signaling pathway. PIC, Vit D, or PIC-Vit D inhibited the expressions of TGF-β, HDAC2, and HDAC4 and inhibited the PI3K/Akt signaling pathway, resulting in lower levels of α-SMA and collagen type I. Image created with BioRender.com (accessed on 29 November 2022).

As explained in Figure 6, the current study’s alternate hypothesis was that PIC, Vit D, or PIC-Vit D would have antifibrotic effects against pulmonary fibrosis through alterations in the levels of expression of HDAC2, HDAC4, and TGF-β in the lungs, and this in turn could inhibit the PI3K/AKT signaling pathway via their antioxidant properties. Pulmonary fibrosis was induced in this study via intratracheal instillation of BLM in rats, and this was consistent with previous studies [30].

TGF-β is a potent inducer of ECM production, including collagen type I and α-SMA [30]. In canonical (SMAD-dependent) pathways, TGF-β activates the expression of profibrotic genes coding for ECM production [31,32]. In non-canonical (SMAD-independent) pathways, TGF-β activates a PI3K/AKT signaling pathway that stimulates the proliferation and differentiation of fibroblasts into myofibroblasts [33]. A previous study showed that fibroblasts from patients with idiopathic pulmonary fibrosis were associated with stimulation of the AKT signaling pathway [34]. Other studies found a correlation between the pathological activation of the PI3K/AKT signaling pathway and the autophagy of alveolar epithelial cells type II (AECIIs) and the production of hydrogen peroxide, which promoted damage of adjacent AECIIs while increasing the proliferation of fibroblasts into myofibroblasts [35,36]. Moreover, TGF-β increased the expression of HDAC2 and HDAC4, resulting in an increase in the expression of ECM proteins such as collagen type I and α-SMA and the proliferation of fibroblasts into myofibroblasts [37]. Previous studies have clearly demonstrated that HDACs can epigenetically control gene expression mediated by TGF-β, resulting in pulmonary fibrosis. Consequently, it was worthwhile to study the possibility that pulmonary fibrosis could be treated by downregulating HDACs [38].

In the current study, intratracheal instillation of BLM upregulated the expression of TGF-β, and this was consistent with previous studies [39]. Treatment with PIC, Vit D, or PIC-Vit D significantly reduced the overexpression of TGF-β in the lungs. Consistent with our results, high TGF-β1 expression in rats with streptozotocin-induced diabetic cardiomyopathy was clearly repressed by oral administration of PIC for 12 weeks. Additionally, PIC displayed anti-inflammatory and hepatoprotective effects in liver fibrosis induced by thioacetamide by repressing TGF-β1 and α-SMA expression, developing MDA, and improving interleukin-10 secretion. A previous experimental study revealed that high hepatic TGF-β1 levels were reduced with the administration of PIC [11]. W. Zhu et al. confirmed that Vit D could alleviate pulmonary fibrosis via a reduction in the expression of TGF-β in the lungs [40].

Upregulated HDAC activities have been revealed in many fibrotic diseases, including those of the lungs, kidneys, liver, and heart. Animal experiments have demonstrated that HDAC inhibitors are beneficial in ameliorating the development and progression of fibrosis [41,42,43,44,45]. In the vast majority of studies, upregulated HDAC activities were accompanied by cell activation, proliferation, differentiation, and anti-apoptosis. Thus, the overexpression of HDACs was associated with various types of cancers [46]. The reports stated that lung fibroblasts obtained from patients with idiopathic pulmonary fibrosis displayed cancer-like characteristics because of the aberrant expression of Class I and Class II HDACs, modifying the acetylation state of chromatin and different non-histone proteins [47].

Overexpression of all class I and II HDACs, particularly HDAC2 and HDAC4, significantly contributed to the progression of pulmonary fibrosis by activating the proliferation of lung fibroblasts into apoptosis-resistant myofibroblasts and increasing the expression of ECM protein-coding genes such as α-SMA and collagen type I [34,48]. It was reported that HDAC2 was crucial for the chronic progression of lung fibrosis, while HDAC4 was crucial for the initial response to lung fibrosis [8,49]. Interestingly, class II HDAC4 has been shown to enhance ECM production in lung myofibroblasts and to participate in profibrotic gene expression by activating the AKT signaling pathway; thus, downregulation of HDAC4 expression inhibited α-SMA expression in normal human lung fibroblasts stimulated by TGF-β1 [8,33,50]. Khalil, W. et al. demonstrated that HDAC4 regulated collagen type I expression via modulation of the antifibrotic levels of miR-29 [51]. On the other hand, class I HDAC2 inhibited tumor suppressor p53 activity via deacetylation of the lysine residues of p53, causing p53 to be unable to bind to DNA and the production of proapoptotic genes to be suppressed [34,52]. Moreover, HDAC2 repressed Fas-mediated apoptosis via the suppression of Fas expression [48].

In the present study, BLM-treated rats exhibited higher levels of HDAC2 and HDAC4 in lung tissues, and this finding agreed with previous studies [48]. Treatment with PIC, Vit D, or PIC-Vit D decreased the expression of HDAC2 and HDAC4 in the lungs, and this in turn suppressed the expression of p-AKT, α-SMA, and collagen type I in fibrotic lungs.

Oxidative stress is considered to be a crucial molecular mechanism underlying pulmonary fibrosis. Ovidative stress is the result of an imbalance between ROS production and the antioxidant defenses, resulting in tissue damage [53]. The lungs have various detoxification pathways for ROS [54]. GSH is one of the non-specific (non-enzymatic) antioxidants that is downregulated in patients with idiopathic pulmonary fibrosis [55,56]. Lipid peroxidation is an oxidation modification of phospholipids in cellular components caused by oxidative stress. MDA is a lipid peroxidation by-product that is increased in the plasma and bronchoalveolar lavage fluid of patients with idiopathic pulmonary fibrosis [55].

In our present study, a challenge with BLM increased oxidative stress, as evidenced by a significant decline in GSH levels and an elevation in the lipid peroxidation by-product MDA. This was consistent with previous studies [57]. Treatment with PIC, Vit D, or PIC-Vit D increased the pulmonary antioxidant capacity by increasing GSH levels and decreasing MDA levels. These findings were in line with those of previous studies, which showed that treatment with PIC could modulate oxidative stress in mice with lipopolysaccharide-induced hepatic endotoxemia by increasing antioxidant enzyme levels and suppressing the MDA levels [58]. PIC also increased the antioxidant capacity and decreased the cellular damage caused by oxidative stress in human periodontal ligament fibroblasts by increasing the GSH levels [59]. P. Yamini et al. confirmed that Vit D decreased neural oxidative stress in sporadic Alzheimer’s disease induced by streptozotocin by increasing GSH levels and decreasing MDA levels [60].

In the present study, pulmonary fibrosis induction was histopathologically manifested by a marked thickening of the alveolar septa, chronic pulmonary inflammation, leukocyte infiltration, emphysematous areas with distortion of the lung architecture, and the marked expansion of fibrous tissue, especially in a peribronchiolar pattern. These fibrotic manifestations were clearly improved by the administration of PIC, Vit D, or PIC-Vit D. W. Zhu et al. confirmed using hematoxylin-and-eosin (H&E) staining that Vit D could inhibit the infiltration of inflammatory cells [40]. H. Sheng et al. confirmed that PIC significantly improved the pathological changes in H&E-stained lung sections from mice [61].

In the current study, immunohistochemical staining of α-SMA was elevated in the BLM group, whereas treatment with PIC, Vit D, or PIC-Vit D led to a significant decline in α-SMA expression in comparison with the BLM group. Similarly, PIC decreased α-SMA expression and pulmonary damage in the BLM group [62]. A previous study found that Vit D deficiency increased α-SMA and collagen type I expression in lung tissues [63]. Immunohistochemical staining in another study revealed that Vit D inhibited the expression of α-SMA in the lungs [40]. This is a preliminary study, and further investigations may be necessary. Multitopic work will be required, followed by clinical trials to approve and recommend the usage of PIC and Vit D for lung fibrosis.

## 5. Conclusions

In summary, our data proved that PIC, Vit D, or PIC-Vit D could suppress pulmonary fibrosis induced by BLM and fibrosis-related gene expression in rats. The antifibrotic effects of PIC, Vit D, or PIC-Vit D may be due to the downregulation of the expression of HDAC2, HDAC4, and TGF-β in lung tissues; suppression of the PI3K/AKT signaling pathway; and antioxidant effects against BLM-induced oxidative damage. The combination of PIC and Vit D improved the beneficial impacts of monotherapy. PIC was superior to Vit D in decreasing the expressions of TGF-β, HDAC2, HDAC4, collagen type I, and MDA in the lungs and in increasing the expression of GSH in the lung; however, PIC was comparable to Vit D in decreasing the expression of t-Akt, p-Akt, and α-SMA in the lungs. PIC has the potential to serve as a molecular target for pulmonary fibrosis therapy in the future. In addition, the utilization of PIC in developing clinical therapeutic strategies could potentially enhance the management of post-COVID-19 pulmonary fibrosis. The advantages of PIC and Vit D usage include a novel approach, multifaceted protection, safety, and the potential for therapeutic intervention. This study served as an initial step for future research endeavors that are necessary to thoroughly investigate the correlation between HDACs and other TGF-β-mediated pathways, as well as the role of other types of HDACs in the development of pulmonary fibrosis. These aspects require further investigation to gain a clearer understanding of them.

## Data Availability

Not applicable.
